# Defect Passivation Using Trichloromelamine for Highly Efficient and Stable Perovskite Solar Cells

**DOI:** 10.3390/polym14030398

**Published:** 2022-01-20

**Authors:** Qiaoli Niu, Ling Zhang, Yao Xu, Chaochao Yuan, Weijie Qi, Shuai Fu, Yuhui Ma, Wenjin Zeng, Ruidong Xia, Yonggang Min

**Affiliations:** 1 Key Laboratory for Organic Electronics and Information Displays, Institute of Advanced Materials, Jiangsu National Synergetic Innovation Center for Advanced Materials (SICAM), Nanjing University of Posts and Telecommunications, 9 Wenyuan Road, Nanjing 210023, China; iamqlniu@njupt.edu.cn (Q.N.); zl13657132936@163.com (L.Z.); xy18936032151@163.com (Y.X.); ycc161217@163.com (C.Y.); qitongxue7@163.com (W.Q.); fs15705186269@163.com (S.F.); 1016061506@njupt.edu.cn (Y.M.); iamwjzeng@njupt.edu.cn (W.Z.); iamygmin@njupt.edu.cn (Y.M.); 2 New Energy Technology Engineering Laboratory of Jiangsu Provence, School of Science, Nanjing University of Posts and Telecommunications, 9 Wenyuan Road, Nanjing 210023, China; 3 The School of Materials and Energy, Guangdong University of Technology, Guangzhou 510006, China; 4 The International School of Advanced Materials, South China University of Technology, 381 Wushan Road, Guangzhou 510640, China

**Keywords:** defects passivation, TCM, perovskite solar cell

## Abstract

Nonradiative recombination losses caused by defects in the perovskite layer seriously affects the efficiency and stability of perovskite solar cells (PSCs). Hence, defect passivation is an effective way to improve the performance of PSCs. In this work, trichloromelamine (TCM) was used as a defects passivator by adding it into the perovskite precursor solution. The experimental results show that the power conversion efficiency (PCE) of PSC increased from 18.87 to 20.15% after the addition of TCM. What’s more, the environmental stability of PSCs was also improved. The working mechanism of TCM was thoroughly investigated, which can be ascribed to the interaction between the –NH– group and uncoordinated lead ions in the perovskite. This work provides a promising strategy for achieving highly efficient and stable PSCs.

## 1. Introduction

Organic-inorganic hybrid perovskite has demonstrated its application potential in the photovoltaic field due to its unique properties, such as its strong light absorption capacity, long charge carrier diffusion length, high charge carrier mobility, and adjustable band gap [1,2,3,4,5]. The power conversion efficiency (PCE) of perovskite solar cells (PSC) has increased from 3.8% to 25.5%, which is comparable to the commercial Si-based solar cell [6]. Although great progress has been made with respect to the performance of PSC, there are still several challenges which remain. One of the key problems is defects in the perovskite films, such as uncoordinated sites (lead and halide ions) [7], lead clusters [8], and vacancy defects [9], which will lead to non-radiative recombination losses. The interaction of these defects with moisture and oxygen will also cause the degradation of the perovskite [10,11,12].

The use of additives for defect passivation is an effective strategy to improve the PCE and stability of PSCs [13,14]. Many types of additives, such as polymers [15], fullerene and its derivatives [16], inorganic acid [17], organic halide salt [18], metal halide salt [19], and nanoparticles [20] were applied to achieve grain boundary (GBs) passivation through morphology control or passivation of crystal defects [21,22,23,24]. Among them, selecting organic molecules with specific functional groups is very effective to improve the PCE and stability of PSCs. For example, the introduction of 3-aminopropyltrimethoxysilane (APMS) inhibited ion migration and passivated defects in perovskite layer because of the interaction between the amino- group and Pb or I ions, leading to the morphology improvement of perovskite film [25]. The PCE of PSC increased from 18.85 to 20.72% [25]. After adding maleimide undecanoic acid (11MA) to the perovskite precursor solution, the density of trap states in perovskite layer reduced due to the strong coordination interaction between 11MA and Pb^2+^, resulting in the increase of PCE from 18.24 to 23.34% [26]. The additive molecules with π-conjugated structure are more likely to gather at the perovskite GBs, inducing the interconnection of the perovskite grains and therefore the more stable PSC [27].

In this work, a low-price organic molecular, trichloromelamine (TCM) was used as a defects passivator in the MAPbI_3_ light-absorbing layer by adding it into the perovskite precursor solution. The chlorine substituted amino groups with Lewis base characteristics in the molecular structure of TCM will simultaneously improve the morphology and passivate defects of the perovskite film. MAPbI_3_ film with improved crystallinity and reduced defect density was obtained. With 0.05 wt% TCM, the PCE of the PSC increased from 18.87% of control device to 20.15%. In addition, after 48 h aging in the dark with 80% humidity at room temperature, the encapsulated device with TCM retained its initial PCE of 77.31%, while the value for the control device is 31.2%. The mechanism was thoroughly investigated, which can be ascribed to the interaction between the –NH– group in TCM and lead ions in MAPbI_3_.

## 2. Materials and Methods

### 2.1. Preparation of NiOx Nanoparticles

First, under magnetic stirring, 12.885 g of NiCl_2_·6H_2_O was dissolved in 100 mL of deionized water. Then, 10 M NaOH solution was added dropwise to the solution until the pH value reached 10. Following, the obtained green solution was centrifuged. After being washed twice with deionized water, the resulting precipitate was dried at 80 °C overnight, and then annealed at 270 °C for 2 h.

### 2.2. Materials and Preparation of Solutions

MAI, PbI_2_ and Pb(Ac)_2_ were purchased from Xi’an Polymer Light Technology Corp, Xi’an, China. [6,6]-phenyl-C61-butyric acid methyl ester (PCBM) and 2,9-Dimethyl-4,7-diphenyl-1,10-phenanthroline (BCP) were purchased from Borun New Material Technology Corp, Ningbo, China.

MAI, PbI_2_, Pb(Ac)_2_ powders were mixed in *N*,*N*-dimethylformamide (DMF, anhydrous, 99.8%, Sigma-Aldrich) solution at a molar ratio of 2.2:0.4:0.6 to prepare MAPbI_3_ precursor solution. Trichloromelamine (TCM) (≥95%, 229.45 (MW), Macklin) was dissolved in DMF and added to the precursor solution at different weight ratios before spin-coating. The weight ratio of TCM varied from 0.01 wt%, 0.05 wt% to 0.1 wt%. PCBM solution (20 mg/mL) was prepared by dissolving it in chlorobenzene (99.5%, Aladding).

### 2.3. Preparation for Characterization

Solutions for NMR test were prepared by adding 0.05 wt/% trichloromelamine into PbI_2_ solution (1 mol/L) in deuterated DMSO.

Perovskite films for XRD, SEM, UV, PL, UPS and XPS tests were all prepared by spin-coating perovskite precursor solution on ITO substrate with a concentration of 1 mol/L. It was spun-coated at 4000 rpm for 30 s. Then the films were thermal annealed on a hot stage at 100 °C for 10 min.

For FT-IR measurement, the above-mentioned perovskite films were scraped from ITO substrates and then blended with spectrum grade KBr. The mixed powder was pressed into pieces before using.

### 2.4. Device Fabrication

Before using, indium tin oxide (ITO) glass substrate (7 Ω, RS^−1^) was sequentially ultrasonically cleaned with detergent, deionized water, acetone, and absolute ethanol for 20 min each. Then, the substrate was cleaned with plasma for 4 min after being dried with a nitrogen stream. Follow that, NiOx nanoparticles dispersion (20 mg/mL in deionized water) was spin-coated at 4000 rpm for 30 s and then annealed at 135 °C for 10 min under atmospheric conditions. Then perovskite precursor solution was spin-coated at 4000 rpm for 30 s in a N_2_-filled glovebox to form perovskite layer, which was heated on a hot plate at 100 °C for 20 min. After that, PCBM film was deposited by spin-coating at 1200 rpm for 30 s. Finally, 5 nm-thick BCP and 100 nm- thick Ag were evaporated as the interface layer and the top metal electrode, under a pressure of 9 × 10^−5^ Pa.

### 2.5. Characterization

Fourier transform infrared (FT-IR) spectroscopy measurements were conducted on a Fourier transform infrared spectrometer (model: IRPrestige-21, range 4000–1000 cm^−1^). Ultraviolet-visible (UV-vis) absorption measurements were measured on a Lamba 35 spectrophotometer (Perkin-Elmer, Waltham, MA, USA). The X-ray diffraction (XRD) patterns of the films were obtained by a Bruker D8 ADVANCE X-ray diffractometer (Bruker Corp, Berlin, Germany) under the operation conditions of 40 kV and 40 mA. The morphology of perovskite films was obtained by field emission scanning electron microscopy (FESEM, S4800 microscope, Hitachi Ltd., Tokyo, Japan). The transient-state photoluminescence (PL) was measure by FLSP920 spectrometer (Edinburgh Instruments Ltd., Livingston, UK). H nuclear magnetic resonance (NMR) spectra were collected by using Bruker DELL PC1 equipment. X-ray photoelectron spectroscopy (XPS) was studied using a PHI Quantera SXM (ULVAC-PHI Inc., Tokyo, Japan). The current density-voltage (*J-V*) curves of the devices were measured by a Keithley 2400 Source Meter under an illumination of 1 sun (100 mW/cm^2^ AM 1.5 G, generated by a solar simulator Oriel Sol3A, Newport Corp., Irvine, CA, USA), which was calibrated with a standard Si photodiode. The active area was 0.096 cm^2^.

## 3. Results

### 3.1. Film Properties

**Figure 1:**
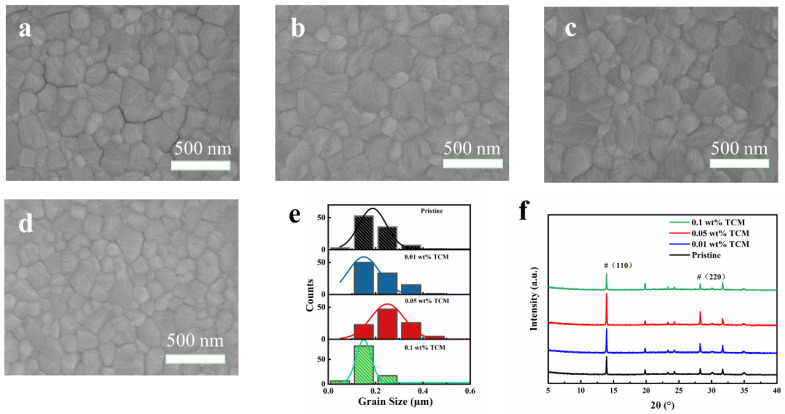
SEM images of perovskite films with different weight ratios of TCM: (**a**) 0%, (**b**) 0.01 wt%, (**c**) 0.05 wt% and (**d**) 0.1 wt%; (**e**) grain size distribution of the perovskite films; (**f**) XRD patterns of perovskite films.

The morphology of the MAPbI_3_ film has great influence on the performance of PSCs. Thus, scanning electron microscopy (SEM) images of MAPbI_3_ films were demonstrated, as shown in Figure 1. The crystal sizes were estimated by using Nano Measurer 1.2 software. For MAPbI_3_ films doped with 0, 0.01, 0.05, and 0.1 wt% TCM, the average grain sizes are 246, 260, 308, and 221 nm, respectively (Figure 1e). Compared with the pristine film, when the doping concentration of TCM increased from 0.01 wt% to 0.1 wt%, the grain size of MAPbI_3_ increased first and then decreased. The MAPbI_3_ film with 0.05 wt% TCM (hereinafter refer to as 0.05 TCM film) has the largest grain size. The effect of TCM on the crystallinity of MAPbI_3_ was studied by X-ray diffraction (XRD) measurement (Figure 1f). All the XRD patterns show significant peaks at 13.90° and 28.17°, which are corresponding to the (110) and (220) planes of MAPbI_3_, respectively. It indicates that all MAPbI_3_ films have orthorhombic crystal structure. Meanwhile, the XRD peak intensity obviously varied after doping TCM, which increased first and then decreased with the concentration increased from 0.01 wt% and 0.05 wt% to 0.1 wt%. The perovskite film with 0.05 wt% TCM has the strongest peak intensity, indicating the best crystallinity. Cross-sectional SEM images of PSCs further proves the crystallinity improvement after adding TCM, as shown in Figure S1. The average thickness of the pristine perovskite layer is 243 nm. It is 260 nm with 0.05 wt% TCM, indicating the larger grain size.

### 3.2. Charge Carrier Dynamic

**Figure 2:**
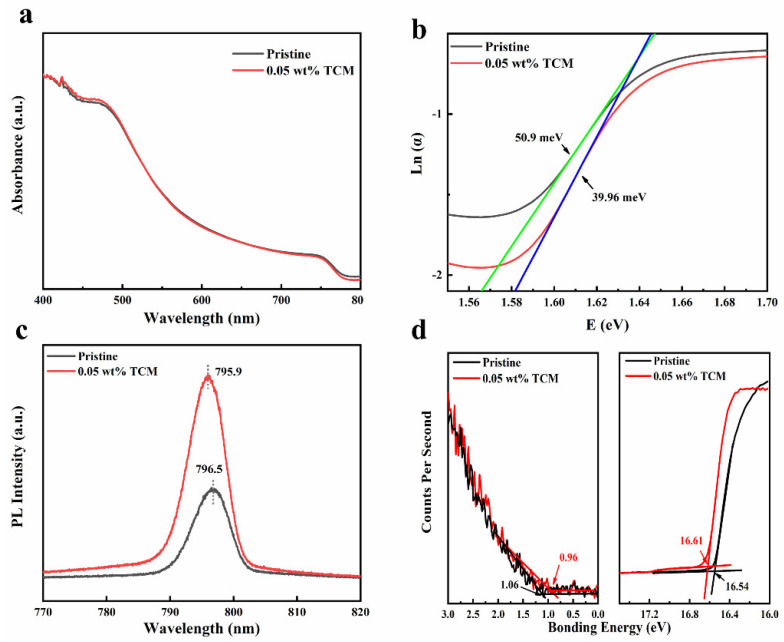
(**a**) Absorbance spectra, (**b**) Urbach energy plot, (**c**) PL spectra, and (**d**) ultraviolet photoelectron spectroscopy (UPS) spectra of MAPbI_3_ films without and with 0.05 wt% TCM.

To investigate the optical properties of MAPbI_3_ films, UV-Vis absorption spectra were measured, as shown in Figure 2a. The same shape of UV-Vis absorption spectra reflects that the crystal structure of MAPbI_3_ film did not change, which is consistent with the XRD results. The absorbance intensity slightly increased at the wavelength lower than 500 nm after adding 0.05 wt% TCM, which can be ascribed to the larger grain size. In addition, for semiconductors, the absorbing edge is called the Urbach tail, which is related to Urbach energy (*E_u_*). Generally, the Urbach energy is the tail width of the local defect state in the band gap, which can be calculated by fitting the exponential part of the Urbach tail according to Equation (1) [28]:

(1) $$\alpha \left(E\right)=\alpha_{0}*\exp\left[\frac{E-E_{0}}{E_{u}}\right]$$

where *α* is the absorption coefficient and *E* is the photon energy [29,30]. *E_u_* value can be calculated by plotting Ln(α) against *E*, as shown in Figure 2b. The calculated *E_u_* values are 50.9 meV and 39.96 meV for the pristine and 0.05 TCM MAPbI_3_ films, respectively. The lower *E_u_* value indicated the reduced defect density. It means that the adding of 0.05 wt% TCM reduced the defect density of MAPbI_3_ film. It was further proved by measuring the steady-state photoluminescence (PL) spectra, as shown in Figure 2c. The PL intensity of MAPbI_3_ film is significantly enhanced with the addition of 0.05 wt% TCM, indicating the reduction of nonradiative recombination losses (which is always caused by defects). In addition, after the addition of TCM, the PL peak blue-shifted from 796.5 nm to 795.9 nm, which can be ascribed to the reduction of surface defects [31]. Therefore, the surface defects of MAPbI_3_ film were passivated by adding TCM, which can also be proved by the Ultraviolet Photoelectron Spectroscopy (UPS) data, as shown in Figure 2d. The Fermi energy level (*E_F_*) with respect to the valance band maximum (VBM) of perovskite films shift from 1.06 to 0.96 eV (Figure 2d) after the introduction of TCM, indicating that it is closer to the center of the bandgap (*E*_g_: 1.55 eV for MAPbI_3_). Thus, the perovskite is more like the intrinsic semiconductor after the passivation of surface defects by TCM [32]. The above experimental results show that the defects of the perovskite film reduced after adding 0.05 wt% TCM.

**Figure 3:**
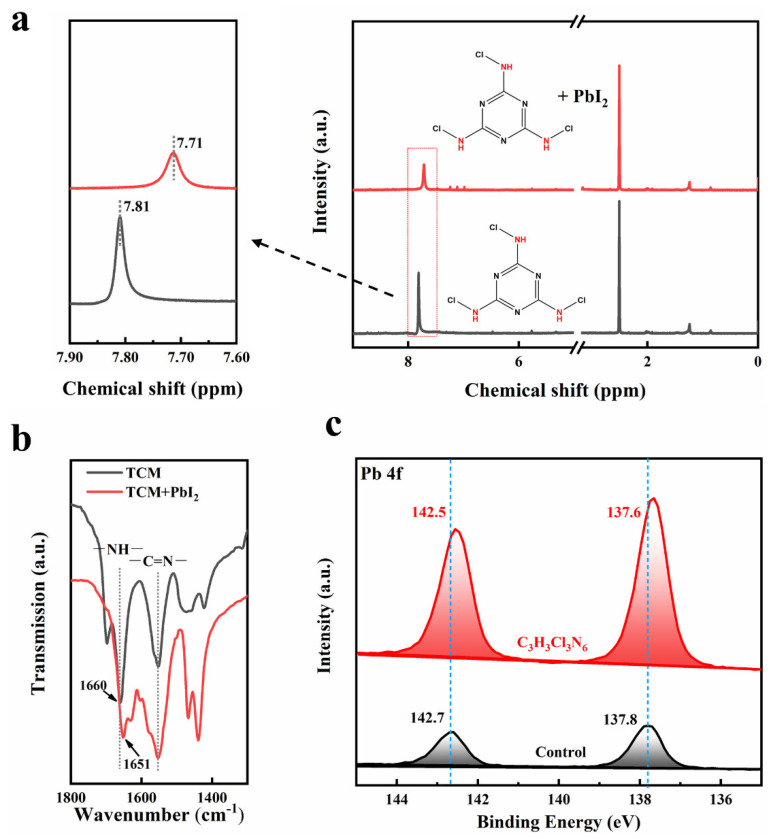
(**a**) ^1^H NMR spectra and (**b**) FT-IR spectra of pure TCM and the mixture of PbI_2_ and TCM, (**c**) XPS spectra for Pb 4f of perovskite with and without TCM.

In order to explore the work mechanism of defect passivation, ^1^H nuclear magnetic resonance (NMR) and Fourier transform infrared spectroscopy (FT-IR) tests were conducted. Figure 3a shows the ^1^H NMR spectra of pure TCM and the mixture of PbI_2_ and TCM. After adding PbI_2_, the chemical shift of the hydrogen peak corresponding to the –NH– group of TCM shifted from 7.81 to 7.71 ppm. It indicates that Pb^2+^ interacted with the –NH– group in TCM. FT-IR was also used to detect the interaction between the –NH– group of TCM and Pb^2+^ (Figure 3b and Figure S2). For pure TCM, the characteristic peak of the stretching vibration of the –NH– group located at 1660 cm^−1^, which is 1651 cm^−1^ for the mixture of TCM and PbI_2_. The shift of the peak position indicates the interaction between the –NH– group and Pb^2+^. X-ray photoelectron spectroscopic (XPS) pattern (Figure 3c) shows that the Pb 4f_7/2_ and Pb 4f_5/2_ signals shift from 137.8 eV and 142.7 eV to 137.6 eV and 142.5 eV, respectively, after the addition of TCM. It demonstrated that the binding energy of the Pb 4f peak reduced by 0.2 eV, which indicates the decreased cationic charge of under-coordinated Pb^2+^ [33].

**Figure 4:**
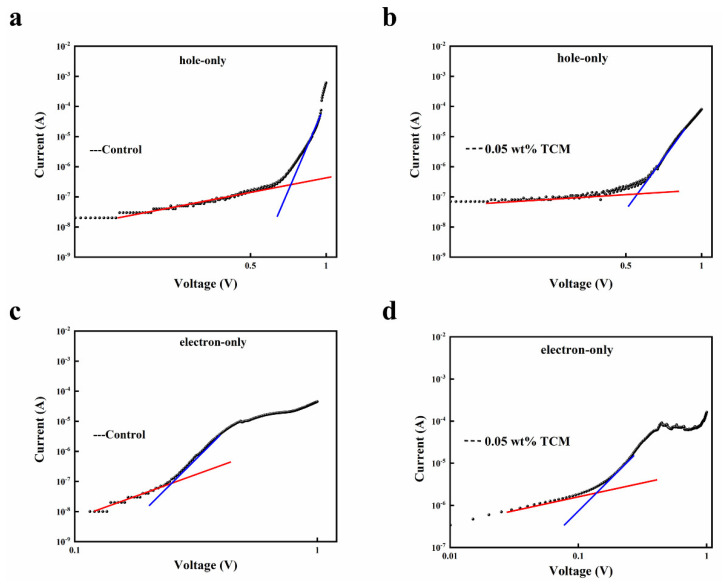
*J-V* curves of hole-only devices: (**a**) control and (**b**) with 0.05 wt% TCM, and electron-only devices: (**c**) control and (**d**) with 0.05 wt% TCM.

To estimate the defect of density (*N_defects_*) of perovskite films, electron-only and hole-only devices with the structure of ITO/SnO_2_/MAPbI_3_/PCBM/Ag and ITO/NiOx/MAPbI_3_/PTAA/Ag were fabricated, respectively. The *J-V* curves are shown in Figure 4. The density of the defects can be calculated in the space-charge-limited current region by Equation (2):

(2) $$N_{defect}=\frac{2\varepsilon \epsilon_{0}V_{TFL}}{eL^{2}}$$

where *ε* is the relative dielectric constant [34], *ε_0_* is the vacuum permittivity, *L* is the thickness of the perovskite film, *e* is the unit charge, and *V_TFL_* is the trap-filled-limit (TFL) voltage. *V_TFL_* refers to the voltage at the kink point from linear region to the TFL region. The *N_defects_* and *V_TFL_* values are summarized in Table 1. It can be seen that the density of the electronic defect of the control device is about twice that of the device with 0.05 wt% TCM. The density of the hole defect states are only slightly reduced (from 2.8 × 10^16^ to 2.2 × 10^16^ cm^−3^). Combined with the results of UPS, this means that the addition of TCM effectively passivated the *n*-type undercoordinated Pb^2+^ defects on the surface of the perovskite film. Therefore, after the addition of TCM, the surface of the perovskite film is more intrinsic. This is consistent with the passivation mechanism of TCM doping described above.

### 3.3. Device Characterization

**Figure 5:**
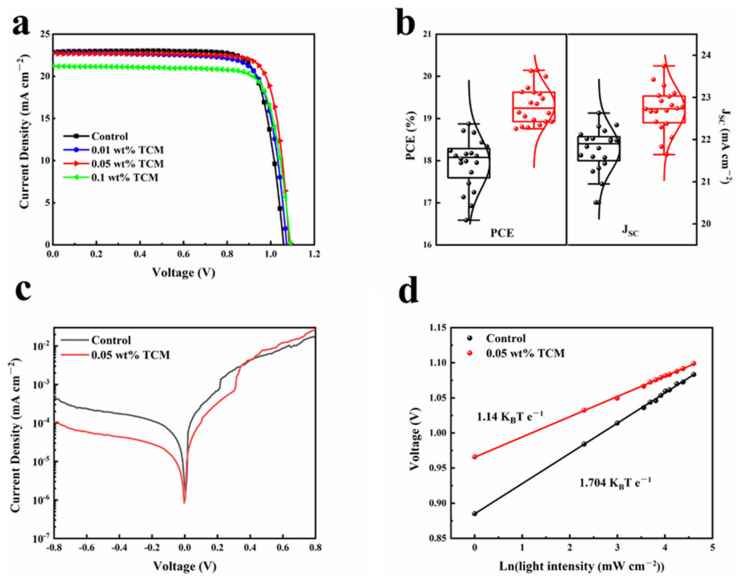
(**a**) *J-V* curves for PSCs with different amounts of TCM additives; (**b**) performance statistic of the PSCs; (**c**) *J-V* curves of PSCs under dark. (**d**) Light intensity dependence of *V*_OC_ of the pristine and 0.05 wt% TCM perovskite films. The light intensity increased from 1 to 100 mW/cm^2^.

Figure 5a show the *J-V* curves of the PSCs, ITO/NiOx/MAPbI_3_/PCBM/Ag (Figure S4) based on MAPbI_3_ with/without TCM. The corresponding performance values are summarized in Table 2. The experimental error values were obtained by subtracting the average value and then divided the results by two. The control device shows a short circuit current density (*J*_SC_) of 22.63 ± 0.43 mA cm^−2^, an open circuit voltage (*V*_OC_) of 1.067 V and a fill factor (*FF*) of 78.14 ± 0.72%, resulting in a PCE of 18.87 ± 0.46%. With the addition of TCM, the performance of PSCs depended on the ratio of TCM. As the weight ratio of TCM increased (*V*_OC_), *FF* and PCE both increased and reached 0.05 wt%. Then, when it continued to increase to 0.1%, *J*_SC_ had a significant drop. Thus, 0.05 wt% is the optimized weight ratio of TCM, based on which PSC with a *V*_OC_ of 1.085 V, a *J*_SC_ of 22.68 ± 0.28 mA cm^−2^, a *FF* of 81.81 ± 1.66%, and therefore a PCE of 20.15% ± 0.41% was obtained. Obviously, the increase of PCE is mainly caused by the enhancement of *V*_OC_ and *FF*, which may benefit from the film quality improvement of the perovskite layer.

The statistics of the photovoltaic performance parameters of PSC (Figure 5b) shows that the 0.05% TCM device not only has higher photovoltaic parameters than the control device but also shows that the distribution is more concentrated. It indicates that after adding TCM, the repeatability of the device is also improved. In addition, as shown in Figure S3, for the control device and device with 0.05 wt% TCM, the forward scanned *J-V* curve matched well with that from reverse scan, indicating a negligible hysteresis effect.

The current density of the 0.05 wt% TCM PSC under dark decreases at low voltage, as shown in Figure 5c. It further proves that the defects density of perovskite film with 0.05 wt% TCM reduced [35], which is conducive to the transfer of charge. Therefore, the series resistance (*Rs*) of the 0.05 wt% TCM device decreased from 50.28 Ω·cm^−2^ of the control device to 29.57 Ω·cm^−2^, as shown in Table 2. The reduction of *R*s value is also responsible for the increase in the *FF* of PSC.

In order to further explore the electrical and optical properties of TCM doped perovskite films, the light intensity dependence of *V*_OC_ had been investigated. It can provide more detailed information about the reorganization process under open circuit conditions according to the following Equation (3) [36,37]

(3) $$\delta V_{oc}=n\left(\frac{k_{B}T}{e}\right)Ln\left(I\right)+\mathrm{constant}$$

where *n* is the ideality factor, *k_B_* is the Boltzmann constant, *q* is the elementary charge, *T* is the absolute temperature, and *I* is the light intensity. The 0.05 wt% TCM device exhibits a higher *V*_OC_ than the control device under the same light intensity (Figure 5d). A plot of *V*_OC_ as a function of logarithmic light intensity *Ln*(*I*) is linearly fitted to evaluate the slope, *n* (*k_B_T/e*), which represents the recombination process caused by the trap states in the optoelectronic device. The slope value of the 0.05 TCM device is 1.14 *k_B_T/e*, which is 1.704 *k_B_T/e* for the control device. The decrease in the slope value means that TCM doping can effectively reduce the trap-assisted carrier recombination, which is consistent with the above results.

**Figure 6:**
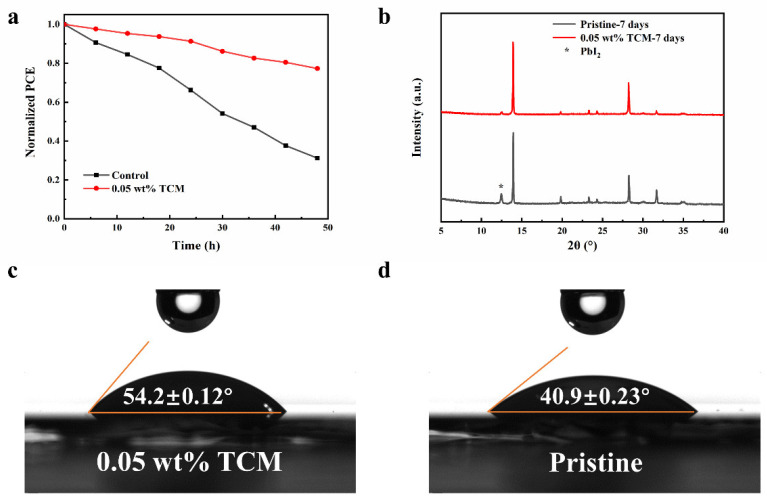
(**a**) Normalized PCE values during stored under 80% humidity in the dark at room temperature, (**b**) XRD patterns of perovskite films aged for seven days, and contact angle of water on perovskite films: (**c**) 0.05 TCM MAPbI_3_ film and (**d**) pristine MAPbI_3_ film.

In order to evaluate the environmental stability of PSCs, the PCE of PSCs were measured during storage. Figure 6a shows the normalized PCE against time. After being stored under 80% humidity in the dark at room temperature for 50 h, the PCE of the 0.05 wt% TCM device retained 77.3% of its initial value, while the value for the control device is 31.2%. Obviously, the 0.05 wt% TCM device is more stable than the control device. XRD was also used to detect the stability of perovskite film after being exposed to ambient air with a humidity of 80% in the dark for a week. As shown in Figure 6b, the peak at 12.8° is observed for both film, which is corresponding to the (100) plane of PbI_2_. Meanwhile, in the XRD pattern of the newly fabricated MAPbI_3_ film (Figure 1f), there was no peak at 12.8°. The precipitation of lead iodide after storage indicates the decomposition of MAPbI_3_. Meanwhile, the peak intensity at 12.8° for the 0.05 TCM MAPbI_3_ film is much lower than that of the pristine film, indicating less MAPbI_3_ decomposed. It means that TCM additive effectively protected the perovskite film from moisture. The contact angle of water on the perovskite film was measured, as shown in Figure 6c–d. It shows that the 0.05 TCM MAPbI_3_ film is more hydrophobic than the pristine film (54.2° vs. 40.9°), which is also responsible for the improved environment stability of the perovskite film and PSCs.

## 4. Discussion

In conclusion, TCM was used as an additive in the MAPbI_3_ film by doping it in the perovskite precursor solution. The experimental results show that TCM passivated the defects at the surface of the MAPbI_3_ film, resulting in the PCE of PSC increasing from 18.87% to 20.15% due to the enhancement of *V_OC_* and *FF*. With TCM, both the PSC and MAPbI_3_ film show improved environmental stability. Passivation is caused by the interaction between the uncoordinated lead ion and the –NH– group in TCM. This work provides an effective method to improve the efficiency and stability of PSCs.

## Figures and Tables

**Table 1:** Summary of *V_TFL_* and *N_defects_* values.

Device	*V_TFL_* (V)		*N_defects_* (cm^−3^)	
	Hole-Only	Electron-Only	Hole-Only	Electron-Only
control	0.724	0.266	2.8 × 10^16^	1.05 × 10^16^
with 0.05 wt% TCM	0.564	0.138	2.2 × 10^16^	5.42 × 10^15^

**Table 2:** Summary of detailed performances parameters of PSCs.

TCM (wt%)	Scan Direction	*V_OC_* (V)	*J_SC_* (mA cm^−2^)	*FF* (%)	PCE (%)	*R*s (Ω cm^−2^)
0	forward	1.070	23.00 ± 0.62	76.46 ± 1.32	18.81 ± 0.31	55.98
	reverse	1.067	22.63 ± 0.86	78.14 ± 1.43	18.87 ± 0.53	50.28
0.01	forward	1.080	22.77 ± 0.57	77.81 ± 0.65	19.13 ± 0.52	43.75
	reverse	1.073	22.75 ± 0.14	78.13 ± 0.43	19.07 ± 0.15	37.66
0.05	forward	1.090	23.42 ± 0.12	78.32 ± 1.31	19.99 ± 0.45	32.97
	reverse	1.085	22.69 ± 0.07	81.81 ± 1.32	20.15 ± 0.13	29.57
0.1	forward	1.090	21.73 ± 0.65	77.59 ± 1.97	18.37 ± 0.58	50.32
	reverse	1.091	21.20 ± 0.52	79.53 ± 0.64	18.39 ± 0.42	45.27

## Data Availability

Data sharing not applicable.

## Supplementary Materials

The following are available online at https://www.mdpi.com/article/10.3390/polym14030398/s1. Figure S1: Cross-sectional SEM images of perovskite devices: (a) control and (b) 0.05 TCM additive; Figure S2: FT-IR spectrum of pure TCM and PbI_2_ mixed with TCM; Figure S3: *J-V* curve of forward and reverse voltage scanning of the best control device and 0.05 TCM device; Figure S4: Device configuration of inverted perovskite solar cells.
