# Supplementary material for: Defect Passivation Using Trichloromelamine for Highly Efficient and Stable Perovskite Solar Cells
Source: Polymers (Basel). 2022 Jan 20;14(3):398. doi: 10.3390/polym14030398 (PMC8839287; doi:10.3390/polym14030398)
Supplement: Supplementary file 1 [file polymers-14-00398-s001.zip › polymers-1519611-supplementary.pdf]

Supporting Information

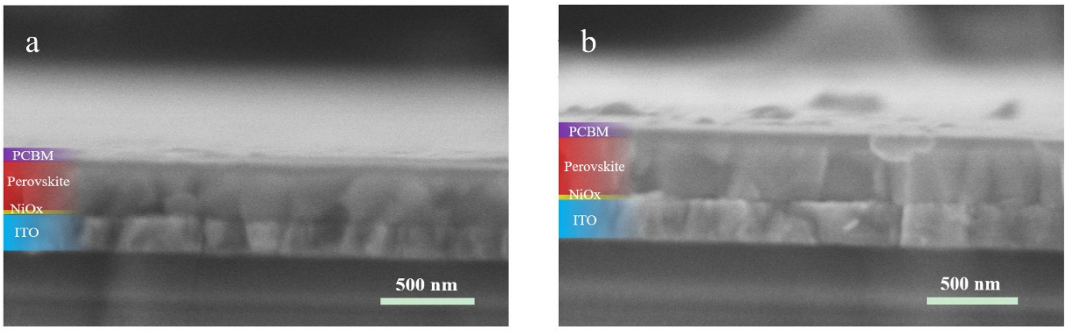

Figure S1. Cross-sectional SEM images of perovskite devices: (**a**) control and (**b**) 0.05 TCM additive.

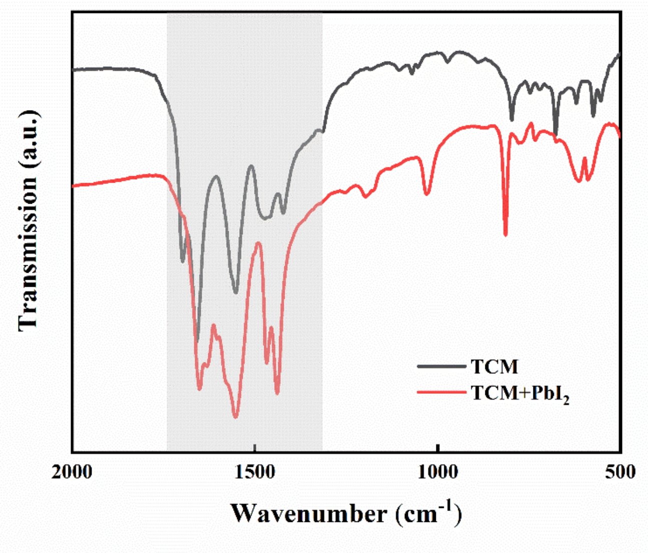

Figure S2. FT-IR spectrum of pure TCM and PbI_2 mixed with TCM.

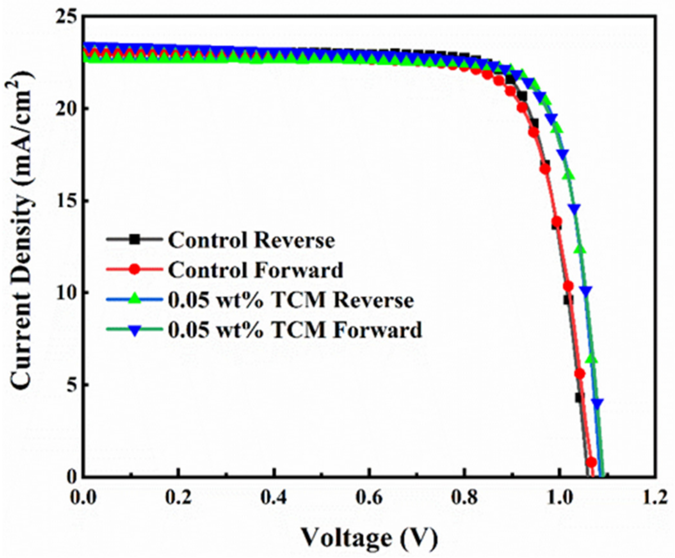

Figure S3. *J-V* curve of forward and reverse voltage scanning of the best control device and 0.05 TCM device.

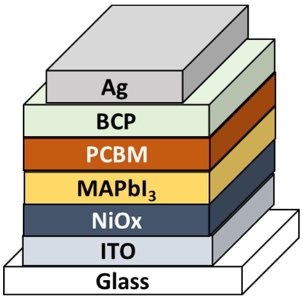

Figure S4. Device configuration of inverted perovskite solar cells
